# Comparative Analysis of SWIRM Domain-Containing Proteins in Plants

**DOI:** 10.1155/2012/310402

**Published:** 2012-07-17

**Authors:** Yan Gao, Songguang Yang, Lianyu Yuan, Yuhai Cui, Keqiang Wu

**Affiliations:** ^1^Key Laboratory of Plant Resources Conservation and Sustainable Utilization, South China Botanical Garden, Chinese Academy of Sciences, Xingke Road 723, Guangzhou 51065, China; ^2^Graduate University of Chinese Academy of Sciences, Chinese Academy of Sciences, Beijing 100049, China; ^3^Agriculture and Agri-Food Canada, Southern Crop Protection and Food Research Centre, London, ON, Canada N5V 4T3; ^4^Institute of Plant Biology, National Taiwan University, Taipei 106, Taiwan

## Abstract

Chromatin-remodeling complexes affect gene expression by using the energy of ATP hydrolysis to locally disrupt or alter the association of histones with DNA. SWIRM (Swi3p, Rsc8p, and Moira) domain is an alpha-helical domain of about 85 residues in chromosomal proteins. SWIRM domain-containing proteins make up large multisubunit complexes by interacting with other chromatin modification factors and may have an important function in plants. However, little is known about SWIRM domain-containing proteins in plants. In this study, 67 SWIRM domain-containing proteins from 6 plant species were identified and analyzed. Plant SWIRM domain proteins can be divided into three distinct types: Swi-type, LSD1-type, and Ada2-type. Generally, the SWIRM domain forms a helix-turn-helix motif commonly found in DNA-binding proteins. The genes encoding SWIRM domain proteins in *Oryza sativa* are widely expressed, especially in pistils. In addition, *OsCHB701* and *OsHDMA701* were downregulated by cold stress, whereas *OsHDMA701* and *OsHDMA702* were significantly induced by heat stress. These observations indicate that SWIRM domain proteins may play an essential role in plant development and plant responses to environmental stress.

## 1. Introduction

In eukaryotes, the genetic information encoded by DNA is packaged into chromatin. The reversibly dynamic changes in chromatin structure modulate the access of regulatory factors to DNA [1, 2]. The precise coordination and organization of chromatin modifications are essential for the correct spatial and temporal maintenance of the epigenetic code within the eukaryotic genome [3, 4]. These changes in chromatin involve activities of many chromatin-modifying complexes,  consisting of both catalytic and noncatalytic subunits [5]. Such subunits are characterized by specific structural frames that mediate protein-protein and protein-DNA interactions. Generally these specific function domains are conserved through evolution. At present, some of these conserved chromosomal protein modules are well studied, such as the bromodomain, the chromodomain, and the SANT domain. Bromodomains were discovered to function as acetyl-lysine binding domains [6, 7]. Chromodomains were commonly found in proteins associated with the remodeling and manipulation of chromatin, mediating specific interactions with proteins and RNA by recognizing lysine methylation in histone tails [8, 9]. SANT domains tether to both DNA and proteins and are essential for histone acetyltransferase activity [10, 11]. Another such conserved domain is the SWIRM identified in several remodeling and modifying complexes.

SWIRM domain was named after the proteins Swi3p, Rsc8p, and Moira, in which it was first recognized. The computational sequence-profile analysis indicates that the typical SWIRM domain consists of 85 amino acid residues and forms a compact helix-turn-helix (HTH)-related structure [12]. Based on the domain architectures and the amino acid sequence homology, the SWIRM domains can be classified into three main types: Swi3/MYSM1 (human MYb-like, Swirm, and Mpn domain-containing protein-1), LSD1 (Lysine-specific demethylase 1), and Ada2 (Adenosine deaminase isoenzymes 2) types [13]. Swi3p-type SWIRM domain-containing proteins are homologous to the ATP-dependent chromatin remodeling complexes SWI/SNF. LSD1-type SWIRM domain-containing proteins belong to Lysine-specific demethylase. LSD1 is the first histone demethylase discovered, and it belongs to the superfamily of the flavin adenine dinucleotide (FAD)-dependent amine oxidases [14, 15]. Ada2-types SWIRM domain-containing proteins are homologs of transcriptional adaptor ADA2a, which promotes histone lysine acetylation and transcriptional activation and acts as a molecular scaffold within the SAGA remodeling complex [16]. 

Recent studies indicate that plant SWIRM domain-containing proteins function in various plant physiological and developmental processes. In* Arabidopsis, *there are four variants of Swi3-type proteins. *AtSWI3A* and *AtSWI3B *are essential for early embryonic development, whereas *AtSWI3C* and *AtSWI3D *affect different phases of vegetative and reproductive development [17]. The *swi3b* mutants display a reduced sensitivity to ABA-mediated inhibition of seed germination and growth and reduced expression of the ABA-responsive genes [18]. Furthermore, AtSWI3B can interact with FCA, a regulator of flowering time in* Arabidopsis *[19]. AtSWI3C is a core subunit of a BRM ATPase-associated SWI/SNF complex [20]. In addition, the LSD1-type SWIRM domain-containing protein AtFLD is involved in the floral transition and regulates the reproductive competence of the shoot [21, 22]. 

Although these studies have provided important insights about SWIRM domain-containing proteins in *Arabidopsis thaliana*, the knowledge on their functions is still scarce in the other plants. In this study, we identified 67 SWIRM domain-containing proteins from 6 plant species including *Arabidopsis thaliana*, *Medicago truncatula*, *Oryza sativa*, *Physcomitrella patens*, *Populus trichocarpa,* and *Zea mays*. The SWIRM proteins from *Oryza sativa* were further characterized and their expression patterns were analyzed.

## 2. Materials and Methods

### 2.1. Identification of the SWIRM Family Proteins in Plants

To identify the SWIRM family proteins in plants, BLAST [23] searches of the NCBI databases (http://www.ncbi.nlm.nih.gov/) were performed using the amino acid sequence of the SWIRM domain in AtSWI3A as a query sequence. All predicted SWIRM domain-containing proteins were used for similarity searches again to confirm these predicted proteins and detect new candidates. The following databases were used in this search: TAIR (The Arabidopsis Information Resource, http://www.arabidopsis.org/), RAPDB (Rice Genome Annotation Project Database and Resource, http://rice.plantbiology.msu.edu/), ProFITs of maize (http://bioinfo.cau.edu.cn/ProFITS/index.php/), and Moss Genome (http://www.mossgenome.org/). We obtained those sequences whose *E* values were below 1*e* ^−5^ and redundant sequences with different identification numbers and the same chromosome loci were removed from our dataset. Amino acid sequences of SWIRM domain-containing proteins were obtained from NCBI and reconfirmed using the Chromatin Database (http://www.chromdb.org/) and respective databases (TAIR, TIGR, RAPDB, and ProFITs of maize).

### 2.2. Phylogenetic Analysis and Multiple Sequence Alignment

Phylogenetic analysis was performed with the MEGA 4.0 program [24] by the neighbor-joining method. Bootstrap analysis was carried out with 1000 replicates based on the complete amino acid sequences. Amino acid sequences of SWIRM domain in fasta formats were used for multiple sequence alignment by ClustalX [25] and then adjusted manually using the GeneDoc software [26].

### 2.3. Bioinformatics Analyses

Conserved domains of the SWIRM family proteins were explored by using the following databases: Pfam (http://pfam.janelia.org/), SMART (http://smart.embl-heidelberg.de/), and CDD (http://www.ncbi.nlm.nih.gov/Structure/cdd/cdd.shtml). The protein secondary structure was predicted by Jpred (http://www.compbio.dundee.ac.uk/www-jpred/) and PSIPRED (http://bioinf.cs.ucl.ac.uk/psipred/). The protein conserved domains were draw by DOG2.0 software [27].

### 2.4. Expression Analysis of OsSWIRMs

The expression patterns of all SWIRM domain-containing proteins were individually queried using Genevestigator (https://www.genevestigator.com/gv/plant.jsp). The expression levels of *OsSWIRMs* in the developmental stages, different anatomical parts and abiotic stress were analyzed. 

## 3. Results

### 3.1. Phylogenetic Analyses of SWIRM Domain-Containing Proteins in Plant

**Figure 1:**
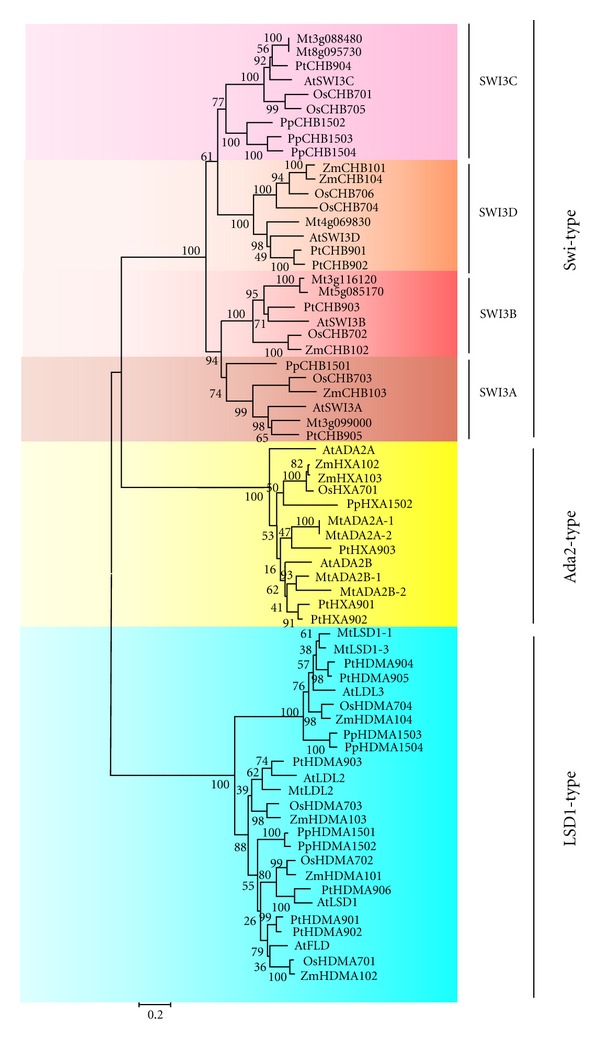
Phylogenetic analysis of SWIRM domain-containing proteins in plants. The neighbor joining phylogenetic tree constructed by MEGA4 summarizes the evolutionary relationships among the 67 members of the SWIRM domain-containing proteins from *Arabidopsis thaliana *(At)*, Medicago truncatula *(Mt)*, Oryza sativa *L. ssp. *Japonica* (Os),* Physcomitrella patens *(Pp)*, Populus trichocarpa *(Pt), and *Zea mays *(Zm)*. *

The amino acid sequence of the AtSWI3A SWIRM domain was used as a query sequence to perform independent searches in NCBI database, then 67 SWIRM domain-containing proteins were identified from *Physcomitrella patens* (lower plant), *Oryza sativa *L. ssp. *Japonica* and *Zea mays* (monocot), *Medicago truncatula *and *Arabidopsis thaliana* (dicot), and *Populus trichocarpa *(xylophyta) (Table 1). To further investigate the evolutionary history of SWIRM domain-containing proteins in plants, we carried out phylogenetic analyses with the 67 amino acid sequences using MEGA 4.0 program. The phylogenetic tree (Figure 1) indicates that the 67 SWIRM domain-containing proteins fall into three clades: Swi3-type subfamily, LSD1-type subfamily, and Ada2-type subfamily, nevertheless, MYSM1-type SWIRM-containing proteins were not found in plants. Considering the structural resemblance between the Swi3 and LSD1 SWIRM domains, it is possible that the Swi3-type and Ada2-type SWIRM proteins had diverged after the appearance of the LSD1-type SWIRM. The plant SWI3 homologues can be clearly divided into four groups: SWI3A, SWI3B, SWI3C, and SWI3D, based on four *Arabidopsis* variants. There are six Swi3-type SWIRM proteins in *Oryza sativa*, among which OsCHB703 belongs to the SWI3A group, OsCHB702 belongs to the SWI3B group, OsCHB701 and OsCHB705 belong to the SWI3C group, while OsCHB704 and OsCHB706 belong to the SWI3D group. Like LSD1-like subfamily in *Arabidopsis thaliana*, four LSD1-type SWIRM proteins were identified in *Oryza sativa*. It is noteworthy that there is only one Ada2-type SWIRM protein in *Oryza sativa* or *Physcomitrella patens*, while at least two or more Ada2-type SWIRM proteins in other four species. 

### 3.2. Multiple Sequence Alignments of SWIRM Domains in Oryza sativa

**Figure 2:**
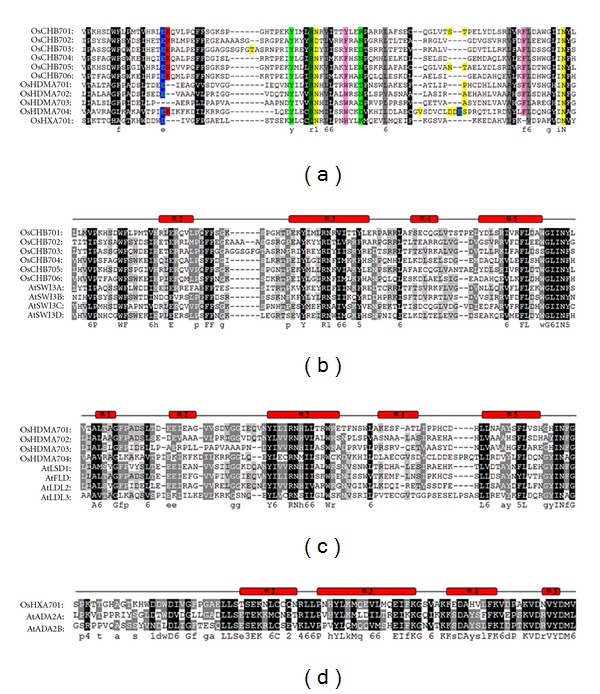
Structure-base sequence alignment of SWIRM domain subtypes. The sequence alignment was produced using Cluatal X. Resulting sequences were then adjusted manually using the GeneDoc software. The secondary structure was predicted by Jpred and PSIPRED. (a) Alignment of the SWIRM domain sequences in *Oryza sativa*. The coloring represents the conservation profile of amino acid residues distinguished by the following amino acid classes: hydrophobic residues are indicated by shaded black, charged residues (ED) by shaded blue, positive residues (RK) by shaded red, polar residues (YNRDTK) by shaded light green, amphoteric residues (RQ) by shaded green, small residues (NDSTPASV) by shaded yellow, aliphatic residues (LIAV) by shaded gray, and aromatic residues (YHWH) by shaded pink. (b) Alignment of the Swi3-type SWIRM domain sequences. The shaded blocks indicate several highly conserved residues by the alignment of SWIRM domain in *Oryza sativa* and *Arabidopsis thaliana*. (c) Alignment of the LSD1-type SWIRM domain sequences. The shaded blocks indicate several highly conserved residues by the alignment of SWIRM domains in *Oryza sativa *and *Arabidopsis thaliana*. (d) Alignment of the Ada2-type SWIRM domain sequences.

To investigate the conserved amino acids of SWIRM domains, we performed multiple sequence alignments of the 11 amino acids sequences of SWIRMs from *Oryza sativa*. These 11 SWIRM domains contain several conserved residues such as polar residues (YNRDTK), amphoteric residues (RQ), small residues (NDSTPASV), aliphatic residues (LIAV), and aromatic residues (YHWH) (Figure 2(a)). 

Generally, the SWIRM domain forms a helix-turn-helix motif commonly found in DNA-binding proteins [28]. Based on the multiple sequence alignments, the secondary-structures of Swi3, Ada2, and LSD1 SWIRM domains in *Oryza sativa* were similar to those in *Arabidopsis* (Figures 2(b), 2(c), and 2(d)). In detail, the SWIRM domain forms a helix-turn-helix-related fold. Swi3-type and Ada2-type SWIRM domains form antiparallel four helical bundles, respectively (Figures 2(b) and 2(d)). However, differing from Swi3, and Ada2-SWIRM, LSD1-SWIRM has an additional helix at its N terminus (Figure 2(c)). 

### 3.3. Analysis of Other Conserved Domains in Oryza sativa

**Figure 3:**
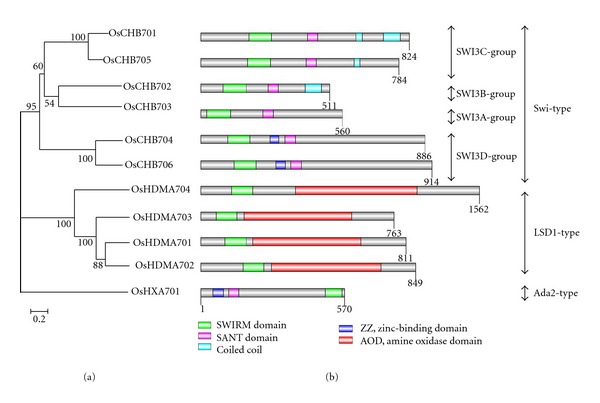
Conserved domains of SWIRM domain-containing proteins in *Oryza sativa. *(a) The neighbor joining phylogenetic tree constructed by MEGA4 summarizes the evolutionary relationships among the 11 members of the SWIRM domain-containing proteins from *Oryza sativa*. (b) The conserved domains of SWIRMs in *Oryza sativa* were drawn by DOG 2.0 with their corresponding amino acid lengths.

Based on the results from Pfam, SMART, and CDD databases, other conserved domains of SWIRM domain-containing proteins were also identified. The results were showed in Figure 3. The structure styles of Swi and Ada-type SWIRM domain-containing proteins are similar. All of them contain SWIRM and SANT domains. SANT domain, also named Myb-like DNA-binding domain, is essential for the *in vivo* functions in the SWI-SNF and ADA complexes. It has been implicated that SANT domain is required for an Ada2p-dependent enhancement of histone tail binding and enzymatic catalysis by Gcn5p [29]. Two members of SWI3D group, OsCHB704 and OsCHB706, have a ZZ zinc finger domain that could also be found in OsHXA701 (Ada2 type). The ZZ-type zinc finger domain, named by its ability to bind two zinc ions, contains 4–6 Cys residues that participate in zinc binding (plus additional Ser/His residues). These zinc fingers are thought to be involved in protein-protein interactions [30]. In addition, OsCHB701 (SWI3C group), OsCHB702 (SWI3B group), and OsCHB703 (SWI3A group) have coiled-coil regions. All of the LSD1-type SWIRM members have an amine oxidases domain (AOD) and a SWIRM domain. Amine oxidases domains are responsible for the demethylase activity through the flavin-adenine-dinucleotide- (FAD-) dependent mechanism (Figure 3(b)) [15]. 

### 3.4. Expression Pattern Analysis of OsSwirms

**Figure 4:**
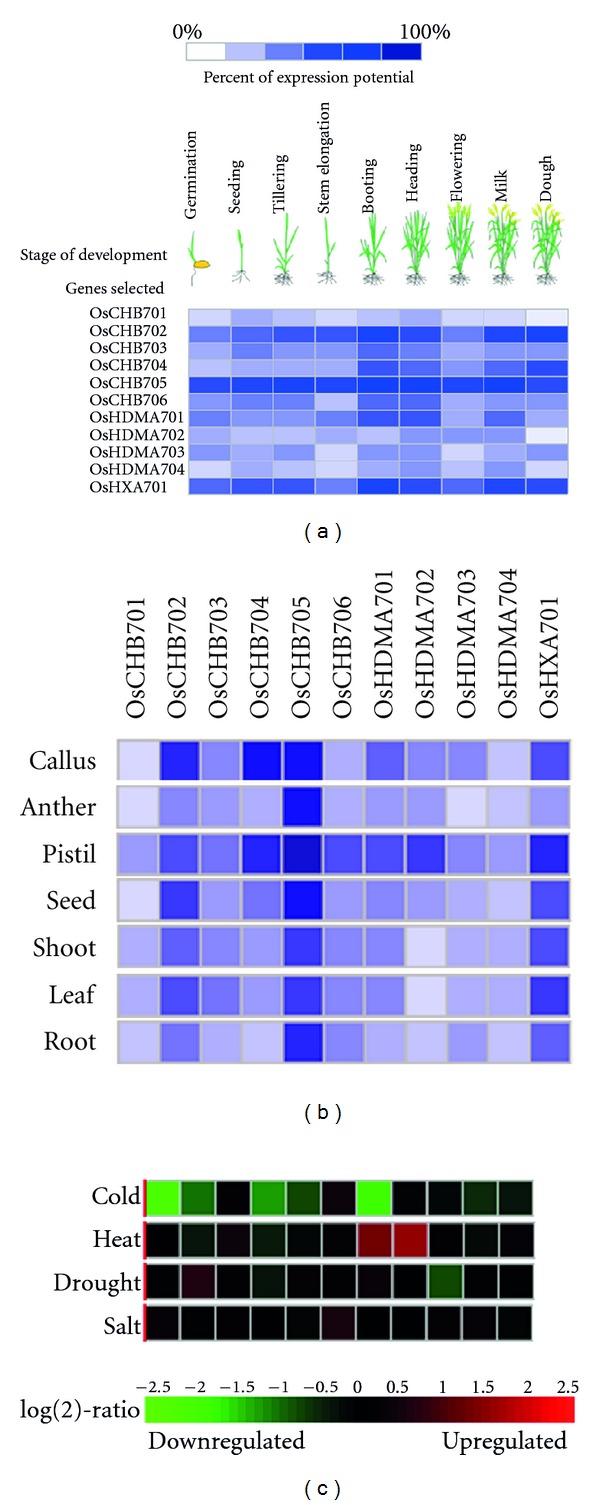
Expression patterns of genes encoding SWIRM family proteins in *Oryza sativa* based on Genevestigator. (a)-(b) Expression patterns of *SWIRM* genes in different developmental stages and different anatomical parts. The darkest blue color represents the maximum level of expression for a given gene across all measurements available in the database for this gene. This means that color intensities can only be compared between elements from the same gene but not with those from other genes. The expression potential of a gene is a robust measure for the maximum of the expression level for this gene. The expression potential is defined as the average of the top 1% signal values across all samples for a given probe set in a given platform. (c) Expression patterns of *SWIRM* genes induced by abiotic stress. Green-red color coding bar represents ratios of treatment versus control values. Gray is used to indicate that both treatment and control are in the background range and that therefore the ratio is not robust. The background threshold *B*(*x*) is a probe set specific expression level. It is defined as *B*(*x*) ≤ 50% quantile of all signals measured of probe set *x* having “absent” calls (*P* value > 0.05) according to MAS5.

Since the function of a gene could be predicted by investigating its expression pattern, we analyzed the gene expression data from Genevestigator. Based on the microarray data, *OsCHB705* keeps a high level of expression almost at all the developmental stages (Figure 4(a)) and in all the tissues (Figure 4(b)). *OsCHB702* and *OsHXA701* share the similar expression profile in developmental process and their highest expression level can be observed at booting, milk, and dough stages (Figure 4(a)). However, *OsCHB702* expresses highest in callus, while* OsHXA701* expresses highest in pistil (Figure 4(b)). In addition, *OsCHB701*, *OsHDMA702*, *OsHDMA703,* and *OsHDMA704* show relatively low expression in all developmental stages (Figure 4(a)) and tissues (Figure 4(b)). Interestingly, compared with the expression level in other tissues, most *OsSWIRMs* show high expression in the pistil (Figure 4(b)). 

The effects of abiotic stresses including cold, heat, drought, and salt on the expression of the 11 *OsSWIRMs* were also investigated. The expression of *OsHDMA701* was significantly down-regulated by cold but up-regulated by heat (Figure 4(c)), implying that *OsHDMA701* was involved in the response to the change in temperature. In addition, the expression of *OsCHB701* was significantly repressed by cold, while the expression of* OsHDMA702* was enhanced by heat (Figure 4(c)). However, the expression of other *OsSWIRMs* was not affected by these abiotic stresses. 

## 4. Conclusion and Discussion

67 SWIRM domain-containing proteins from six plant species were identified and could be divided into three distinct types: Swi-type, LSD1-type, and Ada2-type (Figure 1). No MYSM-type SWIRM domain-containing proteins were found in plants (Figure 1), although they were widely observed in animals [13, 31], indicating that the SWIRM domain-containing proteins may evolve differently between plants and animals. The further analysis suggested that the Swi3-type and Ada2-type SWIRM proteins may diverged after the appearance of the LSD1-type SWIRM (Figure 1). Consistent with the previous reports [12, 32], all of these SWIRM structures in plant form a hHTH-related motif commonly found in DNA-binding proteins and tend to form a long central *α*-helix surrounded by several short helices (Figure 2). In addition, besides the conserved SWIRM domain, each type of SWIRM domain-containing proteins harbored their own distinct motifs, such as SANT, ZZ, and AOD (Figure 3), implying the distinction in function among these proteins. 

There is increasing evidence showing that SWIRM proteins in plants play a crucial role in a range of developmental processes and in responses to abiotic stresses [33]. The expression of the *SWI3C* group* OsCHB701* and *OsCHB705* was not changed appreciably in different developmental stages, while *OSCHB702* and *OsHDMA701* were up-regulated during the booting and heading stage (Figure 4(a)). On the other hand, the expression of Ada2-type SWIRM *OsHXA701* was the lowest in anther, but increased in booting stage and milk stage (Figure 4(a)). The OsCHB702 homolog AtSWI3B was found to interact with FCA, a regulator of flowering time in *Arabidopsis* [19]. In addition, OsHDMA701 homolog AtFLD can interact with CO (CONSTANS) to affect both flowering time and floral initiation in *Arabidopsis *[21, 22]. It remains to be determined whether OSCHB702 and OsHDMA701 are also involved in regulating flower time in *Oryza sativa*. 

*OsCHB702* is highly expressed in callus and seeds. The *atswi3b* mutations resulted in the early embryo lethality [17]. It is possible that *OsCHB702* may also be involved in embryonic and seed development in *Oryza sativa*. Most *OsSWIRMs* are highly expressed in the pistil. In *Arabidopsis*, mutations in *AtSWI3C* and *AtSWI3D *cause aberrant stamen development and abnormal carpel development [17, 20]. These observations suggest that SWI3-type proteins may play a significant role in floral development. Furthermore, *OsCHB701, OsCHB705,* and* OsHDMA701 *can be down-regulated by cold stress, while *OsHDMA701 *and* OsHDMA702* were significantly induced by the heat stress, suggesting a role of these *OsSWIRMs* in abiotic stress response. Our study provides insights into the evolution and function of the plant SWIRM domain proteins. Further studies are required to use functional genetics tools to elucidate clearly the functions of SWIRM domain-containing proteins in different plant species. 

## Figures and Tables

**Table 1:** SWIRM domain-containing proteins in plants.

	Swi3-type subfamily		LSD1-type subfamily		Ada2-type subfamily	Total
*Arabidopsis thaliana*	AtSWI3A AtSWI3C	AtSWI3B AtSWI3D	AtLSD1 AtLDL3	AtLDL2 AtFLD	AtADA2A AtADA2B	10

*Medicago truncatula*		Mt4g069830 (SMARCC1) Mt5g085170 (SMARCC1) Mt3g088480 (SMARCC2) Mt3g099000 (SMARCC2) Mt3g116120 (SMARCC2) Mt8g095730 (SMARCC2)	MtLDL1 MtLDL2 MtLDL3		MtADA2A-1 MtADA2A-2 MtADA2B-1 MtADA2B-2	13

*Oryza sativa* L. ssp. *Japonica *	OsCHB701 OsCHB703 OsCHB705	OsCHB702 OsCHB704 OsCHB706	OsHDMA701 OsHDMA703	OsHDMA702 OsHDMA704	OsHXA701	11

*Physcomitrella patens*	PpCHB1501 PpCHB1503	PpCHB1502 PpCHB1504	PpHDMA1501 PpHDMA1503	PpHDMA1502 PpHDMA1504	PpHXA1502	9

*Populus trichocarpa*	PtCHB901 PtCHB903 PtCHB905	PtCHB902 PtCHB904	PtHDMA901 PtHDMA903 PtHDMA905	PtHDMA902 PtHDMA904 PtHDMA906	PtHXA901 PtHXA902 PtHXA903	14

*Zea mays*	ZmCHB101 ZmCHB103	ZmCHB102 ZmCHB104	ZmHDMA101 ZmHDMA103	ZmHDMA102 ZmHDMA104	ZmHXA102 ZmHXA103	10

Alias and other names referred to the SWIRM domain-containing proteinsin *Arabidopsis thaliana* and *Medicago truncatula* were assigned by http://www.arabidopsis.org/ and http://www.jcvi.org/cgi-bin/medicago/overview.cgi. The names of SWIRM domain-containing proteins in *Oryza sativa*,* Physcomitrella patens*,* Populus trichocarpa, *and *Zea mays *are assigned by chromatin database (http://www.chromdb.org).
